# The Epidemiology and Phenotypes of Ocular Manifestations in Childhood and Juvenile Myasthenia Gravis: A Review

**DOI:** 10.3389/fneur.2022.834212

**Published:** 2022-02-23

**Authors:** Jeannine M. Heckmann, Tarin A. Europa, Aayesha J. Soni, Melissa Nel

**Affiliations:** ^1^Division of Neurology, Department of Medicine, University of Cape Town, Cape Town, South Africa; ^2^Neurology Research Group, University of Cape Town (UCT) Neuroscience Institute, University of Cape Town, Cape Town, South Africa

**Keywords:** treatment refractory ophthalmoplegia, ocular myasthenia gravis (OMG), childhood myasthenia gravis, juvenile myasthenia gravis, genetic susceptibility, Asian ancestry, myasthenia (myasthenia gravis—MG), African ancestry

## Abstract

Myasthenia gravis (MG) appears to have a similar incidence among adult populations worldwide. However, epidemiological and phenotypic differences have been noted among children and juveniles with MG. We reviewed the literature on childhood- and juvenile-onset MG among different populations, with the focus on ocular involvement, antibody profiles, the genetic susceptibility to juvenile MG phenotypes, the use of immune treatments, and the reported responses of extraocular muscles to therapies. Although epidemiological studies used different methodologies, reports from Asia, compared to Europe, showed more than two-fold higher proportions of prepubertal onset (before 12 years) vs. postpubertal-onset juveniles with MG. Compared to European children, ocular MG was 4-fold more frequent among Asian children, and 2–3-fold more frequent among children with African ancestry both in prepubertal and postpubertal ages at onset. These results suggest genetic influences. In Asia, HLA-B^*^46 and DRB1^*^09 appeared overrepresented in children with ocular MG. In Europe, children with MG had a significantly higher rate of transforming from ocular to generalized disease and with an overrepresentation of HLADRB1^*^04. Although treatment regimens vary widely and the responses to immune therapies of the ocular muscles involved in MG were generally poorly described, there were indications that earlier use of steroid therapy may have better outcomes. Reports of treatment-resistant ophthalmoplegia may be more frequent in African and Asian juvenile MG cohorts compared to Europeans. Genetic and muscle gene expression studies point to dysregulated muscle atrophy signaling and mitochondrial metabolism pathways as pathogenetic mechanisms underpinning treatment-resistant ophthalmoplegia in susceptible individuals. In conclusion, phenotypic differences in juveniles with ocular manifestations of MG were evident in different populations suggesting pathogenetic influences. Treatment responses in MG-associated ocular disease should attract more careful descriptive reports. In MG, extraocular muscles may be vulnerable to critical periods of poor force generation and certain individuals may be particularly susceptible to developing treatment-resistant ophthalmoplegia. The development of prognostic biomarkers to identify these susceptible individuals is an unmet need.

## Background

Myasthenia gravis (MG) represents a heterogeneous group of autoantibody-mediated diseases targeting the neuromuscular junction. Extraocular muscles (EOMs) are highly susceptible to manifesting myasthenic weakness and are frequently involved early in the MG disease course prior to developing generalized myasthenia (1, 2). Younger children appear to have a higher prevalence of developing ocular MG (myasthenia remains confined to the EOMs for an extended period) among Asian cohorts, but the outcomes of EOMs to MG therapies are generally not adequately described. This study aimed to review the epidemiological literature of childhood and juvenile MG and determine the severity of ocular phenotypes and treatment responses, as well as current postulates related to the pathogenetic mechanisms underlying the ocular phenotypes with the focus on, but not confined to, the past decade.

## Methods

### Search Strategy and Selection Criteria

#### Epidemiological Data

We searched the PubMed database for reports published in English between January 1, 2010 and October 31, 2021 with the MeSH terms “juvenile” or “childhood” in combination with “myasthenia gravis,” “ocular myasthenia gravis,” and “antibody.” We also selected references from manual searches of reference lists of articles and reviews. Some of these references were published before 2010, but after 1991. We included publications that had clearly stated diagnostic criteria and in which epidemiological data could be extracted such as age at onset and phenotypic characteristics such as acetylcholine receptor (AChR)-antibody status, ocular involvement, secondary generalization, frequency of autoimmune diseases and thymoma, sex differences, and outcomes of ocular myasthenia. If there were 2 publications from the same group, then we included only the most recent article unless unique data was mentioned in the first report. For juvenile MG, we included reports specifying age at onset of MG symptoms between 1 and 20 years, despite the most frequent age cutoff for juvenile onset MG being <18 years (3).

#### Genetic Data

Search terms included; “gene” or “HLA” and “ocular myasthenia,” “juvenile myasthenia gravis,” “childhood myasthenia gravis,” and “extraocular muscles.” We also searched using geographical terms “Asia,” “China,” “Africa,” and “myasthenia gravis.” Original research articles written in English and published between 1996 and October 2021, which compared MG/MG subgroup vs. age and race matched healthy controls, were selected for review particularly if there was a special reference to EOM involvement at presentation, treatment approaches, and descriptive outcomes to treatment. When appropriate data were extracted for positive individual human leukocyte antigen (HLA) associations (excluding haplotypes) with MG (by subgroup if specified).

### Data Extraction and Organization

Although the use of critical appraisal tools to judge the scientific merit of studies for inclusion in a review is encouraged, the scarcity of studies including adequate descriptions of ocular manifestations made the use of such tools difficult to implement. A further limitation was the heterogeneity of age cutoffs for juveniles, childhood, prepubertal and postpubertal cases with MG; while most reports define the age of 12 years as the threshold of puberty and <18 years as juvenile onset, there were different cutoffs to differentiate postpubertal MG from early-onset adult MG, and childhood MG from prepubertal MG. These were indicated as per author(s) and grouped together for comparative purposes (Table 1).

**Table 1:** Characteristics of juvenile myasthenia gravis (MG) and subgroups (pre-pubertal vs. post-pubertal) by race and/or geographical area.

**References**	**Region**		**Pre-pubertal MG**			**Post-pubertal MG**			**JMG**
		**N**	**AAO (%)**	**AChR+**	**OMG**	**AAO (%)**	**AChR+**	**OMG**	**Thymoma**
**Asian and Indian ancestry juveniles**									
Murai et al. (4)	Japan	268	<10	≈50%	62–81%	NR	NR	NR	4–10%
Gui et al. (5)	China	424^*^	≤ 10 (86%)	≈70%	≈95%	10–14 (14%)	≈70%	≈95%	17%
Feng et al. (6)	China South	130	<10	58%	NR	10–19	42%	NR	NR
Lee et al. (7)	South Korea	88	<12 (74%)	90%	97%	12–18 (26%)	87%	70%	NR
Wang et al. (8)	China North	302	<5 (50%)	NR	73%	5–15 (≈50%)	NR	66%	NR
**Cohorts with** **>40% African ancestry juveniles**									
Xu et al. (9)	USA (Texas)	60	<10 (40%)	NR	58%	10–17 (60%)	NR	14%	NR
Barraud et al. (10)	France	40	<12 (48%)	58%	37%	12–18 (52%)	NR	24%	2%
Heckmann et al. (11)	South Africa	190	<12 (41%)	56%	43%	12–20 (69%)	NR	NR	1–3%
**Cohorts with** **>45% European ancestry juveniles**									
VanderPluym et al. (12)	Canada^**^	49	≤ 12 (80%)	52%	46%	13–17 (20%)	≈90%	0	NR
Evoli et al. (13)	Italy	19	<10	74%	26%	NR	NR	NR	0%
Popperud et al. (14)	Norway	63	<12 (33%)	57%	14%^#^	12–18 (67%)	83%	12%	0%
Jastrzebska et al. (15)	Poland	101	<12 (15%)	71%	NR	12–18 (85%)	94%	NR	1%
**Juvenile MG**									
Wong et al. (16)	Hong Kong	101	–	–	–	<16	ND	71%	8%
Chou et al. (17)	Taiwan	54	–	–	–	<20	57%	78%	2%
Ashraf et al. (18)	India	77	–	–	–	<15	^##^	27%	1%
Mansukhani et al. (19)	USA	217	–	–	–	<19	83%	23%	0%
Vecchio et al. (20)	UK	74	–	–	–	<16	84%	51%	NR

*Inclusion into this table required some demographic details according to the columns. AAO refers to the age at symptoms onset (in years as indicated by the respective authors) and % refers to the proportion of the juvenile sample satisfying the prepubertal or postpubertal definition (as indicated for each study) if available; N, refers to sample size; NR, nor reported; ND, not done; OMG refers to ocular MG (for this review, persistence of ocular only symptoms >1 year); JMG, juvenile MG; AChR+ refers to those with detectable antibodies to the acetylcholine receptor*.

* *5 years of follow-up required for inclusion*;

** *48% of cohort European and 28% Asian ancestries; – indicates incomplete data for prepubertal vs. postpubertal, therefore presented data as juvenile MG*.

# *used the follow-up data*.

## *AChR+ data only available for 18% (11/14 AChR+)*.

## Results

### Population Differences in MG by age at Symptom Onset

Although there is recognition worldwide of an increasing predominance of MG among the elderly, including in Asia and Africa (4, 21–25), incidence rates among younger people manifesting with MG appear to differ between Asia and Europe. Population data including children are sparse and methodologies vary widely, but there appear to be four-fold higher incidence rates of MG among younger children from Asia compared to Europe and North America (12, 19, 26). A multiracial pediatric cohort from the United Kingdom (UK) in which data were accrued over 10 years showed similar findings with higher proportions of Afro-Caribbean, Asian, and Arabic children with MG compared to Caucasian children living in the UK (20).

Reports from China regarding the proportions of juveniles with MG, vary substantially and ranged between 27% (302/1,108) in northern China, and 45% (964/2,154) in southern China (27) (Table 1). Nevertheless, at least half of the children manifested with MG before the age of 10, and the incidence peaked in those presenting with symptoms before the age of 5 years (6, 8) (Table 1). A nationwide MG prevalence questionnaire from Japan showed that children developing MG before the age of 10 years accounted for 9% of the overall proportion of MG cases (*n* = 3,061) (4), which is much lower compared to China, but remains substantially higher than the 2% prevalence in Italy (13). Therefore, despite the possible impact of differences in study methodology on the epidemiological results, the incidence of MG in both the prepubertal and postpubertal juveniles, compared to adult-onset disease, was lower in juveniles with European genetic ancestry compared to those with Asian and African genetic ancestry.

### Population and Phenotype Differences Among Categories of Juveniles With MG

#### Prepubertal vs. Postpubertal Onset

There is accumulating evidence that MG presenting in the prepubertal phase in contrast to postpubertal onset differs by genetic ancestry. Studies from Asia showed the proportions of children developing myasthenia before puberty (≥74%) were more than twice as high compared to postpubertal children, and contrasts with a more even distribution (~40 to 48%) amongst cohorts with African children, and <33% in cohorts comprising European children (Table 1). A large cohort from China showed that half of the juveniles developing MG before age 15 were younger than 5 years (8).

In Asia, there was a definite tendency toward more ocular MG amongst the very young, prepubertal children compared to older aged children with MG, but this was not evident in the Norwegian children (Table 1). A multiracial juvenile MG cohort from Canada, in which only 48% had European ancestry, also showed a much higher proportion of prepubertal onset MG, and most of the very young onset ocular MG cases (aged ≤ 6 years) had Asian ancestry (12).

Interestingly, two multiracial cohorts from France (48% of 40 had African ancestry) (10) and the UK (54% of 74 did not have European ancestry) (20) showed similar results in which prepubertal ocular MG were more likely in the African children despite equal proportions of children with pre- and postpubertal MG. A feature of MG among north European children (Norway and Italy) was that ocular only presentations of MG occurred in less than a third, with most children (>75%) developing generalized disease (with/or without respiratory involvement) within 2 years, and between 15 and 26% remained with ocular MG (13, 14). Similar observations were noted in Canada where white children were more likely to develop generalized MG, and Asian children remained with ocular disease (12). Furthermore, the conversion of ocular MG cases to generalized disease was reported in only 5 to 20% of Chinese and Thai children (5, 16, 28, 29) and among 25% of the French cohort in which almost half the children had African genetic ancestry (10).

Sex differences and severity of MG were not consistently different in postpubertal cohorts from different populations; a European cohort showed more girls in the postpubertal group with less severe MG disease (14); two Asian cohorts showed similar proportions of girls and boys, but inconsistent severity of MG grades by sex were reported (7, 28). An older study from the USA, which specifically assessed MG outcomes by race in a clinical setting where the same treatment approaches were used for all children, reported infrequent clinical remissions in prepubertal black patients compared to white patients, although overall disease severity was similar irrespective of race (30). It is important to highlight that MG crises can occur in children and require appropriate immune therapies (3, 12, 26).

In summary, pre- and postpubertal MG cases were more likely to remain confined to the ocular muscles in Asian children compared to those in Europe.

#### Antibody Profile

The AChR-Ab positive MG frequencies by RIA appeared to be similar in all children and in almost all studies ranged between 50 and 95%, irrespective of whether the MG onset was prepubertal or postpubertal (Table 1). A study from China found similar proportions of AChR-Abs by RIA and cell-based assay (CBA) in juveniles (<19 years of age) compared to adult-onset MG cases, although 18% of the juveniles (compared to 10% of adults) were only positive by CBA (31). The age-adjusted incidence rates of AChR-Ab positive MG among juveniles from South Africa (24) appeared to be higher than in Caucasian cohorts from the UK, USA, Norway, and Canada (≈3 per million vs. <1.5 per million, respectively) (12, 19, 32, 33).

Data on the prevalence of muscle-specific kinase (MuSK)-Abs are sparse. Only rare cases of MuSK-Ab positive MG have been reported in juveniles from China [0/118 (31) or <3% (6)], Japan (1.4%) (4), North America (34), and northern Europe (35) and possibly more than expected in the two cohorts with African ancestry children (10, 14, 15, 17, 19, 20).

Overall, most populations reported that younger children were more likely to have AChR-Ab negative MG and ocular disease, both of which conferred a higher likelihood of obtaining remission status (17, 20, 28). However, in the situation where the child does not respond to treatment, despite symptom onset after infancy, the question of possible congenital myasthenia may arise. Clinical features supportive of autoimmune MG include: subacute progressive onset; marked asymmetry of ptosis; substantial fluctuations of ophthalmoplegia (36).

#### Thymoma Incidence

Thymoma occurs rarely in juveniles with MG (34). Data from Asia varied between 0 (0/118) (31) and 17% (6/34) (Table 1).

#### Autoimmune Disease

Concomitant autoimmune disease, mainly thyroid disease, was reported in 4–19% of children with MG from China, Thailand, Hong Kong, and racially diverse cohorts from Canada and the UK (4, 5, 12, 16, 20, 27–29), 27% from Taiwan (included MG onset before age 20) (17) and ≈7% in juvenile cohorts with substantial African ancestry MG cases (10, 11). In contrast, ≈30% of pre- and postpubertal Norwegian children had other autoimmune diseases in addition to MG (14).

### Epidemiology of Ocular MG Among Juveniles

The higher frequencies of ocular MG among younger children from Asia differed substantially from Europe (4, 22, 28) (Table 1). Within the prepubertal onset range, the very young children presenting with symptoms before the age of 4, showed the highest proportions of ocular MG compared to older children from China and Japan (4, 23, 28). African, Afro-Caribbean, and African-American prepubertal onset children also showed higher proportions of ocular MG compared to postpubertal juveniles (9, 11, 12, 20).

### Severity of Extraocular Muscle Involvement at the Presentation of MG in Juveniles vs. Adults

There was a paucity of descriptive data of EOM involvement in MG. An audit of the examination findings in adults presenting with MG to a Scottish ophthalmological service, reported bilateral weakness of multiple EOMs in more than half the patients, irrespective of age, with 6% having bilateral ophthalmopareses (or duction failure) (37). A review from Thailand, but in juveniles (<15 years) presenting with ocular MG, also found limitations of EOM movement in more than 50% (of 62), and most had complete duction failure (29). Juvenile MG cases seen at the Mayo clinic (most were Caucasian children) found limitations of EOM movement in 30%, although there may be a bias to more severe cases in this cohort as most patients were not residents of the county (19).

Observational descriptive EOM data from a largely adult MG clinic, prior to any therapy and in which ≈15% had only ocular manifestations of MG, showed that ≈12% of MG cases had fatigable ptosis/diplopia compared to ≈87% with persistent ophthalmoparesis (or weakness) with or without ptosis in at least one EOM (38). Of those with ophthalmoparesis, > 60% had weakness of ≥ 6 EOMs. There was a trend toward more severe weakness in those with generalized MG compared to ocular only MG (severity is defined by the number of EOMs with ≥ 50% weakness (i.e., can only move half of the EOM's full trajectory) (38). It is worth mentioning that even mild weakness of one EOM may cause diplopia, and those patients with complete ophthalmoplegia may not experience diplopia, although minor malignment of the visual axes may result in diplopia (39).

Taken together, a substantial proportion of patients with MG may develop persistent weakness of their EOMs (ophthalmoparesis or ophthalmoplegia), and this may occur more frequently in juveniles. However, the absence of a standardized approach to reporting does not allow for firm conclusions (see below).

### Treatment Outcomes of Extraocular Muscles in MG

The quantitative and descriptive data with respect to EOM outcomes to therapy in juveniles with MG, were sparse, highlighting a research gap (Table 2). A large cohort of 306 juveniles with ocular MG from southern China, of whom most were treated with immune therapy in addition to pyridostigmine, only 50% achieved minimal manifestations (43) or better after at least 12 months of follow-up (28). Better outcomes were related to earlier use of “standard treatment” (within 2 years of symptom onset), which included the use of prednisone 0.25 mg/kg/day if symptoms did not resolve with pyridostigmine alone, followed by a slow taper and steroid cessation after 6 months of clinical remission (28). Another large study from China, in which 95% of juveniles had ocular involvement, only 17% “improved” while the remainder were either unchanged or worse, despite immune treatments (advising prednisone 0.75 mg/kg/day with poor responses to pyridostigmine), and even thymectomies (5).

**Table 2:** Outcomes of extraocular muscles in juveniles with MG by region.

	**Region**	**AAO, years**	**N**	**Follow-up, years (mean)**	**Ocular outcomes: good vs. treatment resistance as %**	**OMG patients on immune treatment**
Kim et al. (40)	S/Korea	<15	24	3.1	NR; 10% TRO	75%
Lee et al. (7)	S/Korea	<18	88	>2.6	65% vs. 0	>55%
Kraithat et al. (41)	Thailand	<15	14	6.3	93% vs. 7%	79%
Vanikieti et al. (29)	Thailand	<15	62	>4	NR; 8% TRO	52%
Huang et al. (28)	China	<18	306	>1	NR; 50% in remission	93%
Gui et al. (5)	China	<14	424	>5	NR; most unchanged/worse	100%
Ortiz and Borchert (42)	US	<12	21	6.5	NR; OMG resolved in 19%	29%
Xu et al. (9)	US	<18	22	NR	NR; 0 TRO	“Almost all”

*AAO, age at onset; OMG, ocular myasthenia gravis; N, refers to sample size; Ocular outcome: “Good” refers to remission or minimal symptoms and “treatment resistance” refers unchanged or worse; TRO refers to treatment-resistant ophthalmoplegia. S/Korea, South Korea; NR refers to not reported*.

The retrospective results of hospital-based pediatric clinics in South Africa showed, after a median follow-up of 5 years, 31% of prepubertal children (*n* = 31) remained with partial or complete treatment-resistant ophthalmoplegia, and 12% in the postpubertal group (*n* = 20) (11). Although immune treatments were used in this case series, the treatment protocols varied from site to site. In contrast, the pediatric group from North America (*n* = 22; 40% of children with African ancestry) in which >80% were treated within a median of 5 months from symptom onset, and using doses of prednisone 2.5 mg/kg/day for 4–6 weeks before a reduction to alternate day dosing, resulted in all the patients reaching minimal manifestation status or better (9).

In a cohort of predominantly adult MG patients, longitudinal observational data to assess the duration of immune treatment required before the resolution of MG-induced EOM paresis showed that starting immune therapy earlier (<12 months of symptom onset) and using higher doses of prednisone in the first 3 months (0.45 vs. 0.29 mg/kg) associated with significantly better outcomes; patients whose ophthalmoplegia resolved within 3 months of starting therapy had received the higher dose compared to those who only showed resolution of ophthalmoplegia between 4 and 12 months (38). Although there were only nine of 76 patients with MG manifesting with MG before the age of 20 in this cohort, the younger people were less likely to show resolution at 12 months compared to the older people (statistical analyses were not performed due to sample size). Of those with EOM weakness at baseline, 24% remained with complete ophthalmoplegia (all the 12 EOMs with persistent paresis) at 12 months despite moderate doses of prednisone ≈0.35 mg/kg daily with/without steroid-sparing agents (38). These results support the treatment recommendations from Kupersmith and Ying to use earlier and higher doses of prednisone, up to 60 mg daily, for short periods in treating the EOM manifestations of MG (44).

An international working group advising on therapies for juvenile MG recommended starting cholinesterase inhibitors at 0.5 to 1 mg/kg every 4 to 6 h and increasing the dose to 7 mg/kg/day in divided doses for symptom control (3). In our experience, cholinesterase inhibitors may produce some symptomatic relief to the ocular manifestations of MG, especially ptosis, but rarely result in resolution of symptoms; however, others have noted that >50% of patients improve symptomatically on cholinesterase treatment (10). Oral steroids, between 0.5 and 1 mg/kg daily (or 1.5 mg/kg alternate days), are advised in increasing doses in juveniles not responding to cholinesterase inhibitors, with lower doses advised in children with only ocular manifestations (3). Several groups recommend adding steroid-sparing agents to prednisone in children in the setting of poor treatment responses to steroids (3, 5, 11, 12, 28). Steroid-sparing agents which are used in juveniles include azathioprine, mycophenolate mofetil, and rituximab (3). Although methotrexate is increasingly accepted as a cost-effective adjunct to the MG therapeutic armamentarium in adults (45) based on decades of experience in the juvenile arthritides among others, we also use methotrexate in children (10–15 mg/m^2^/week plus folic acid >24 h after methotrexate (folate dose≈ 1/3 of methotrexate) (46).

#### Differential Diagnosis for Treatment-Resistant Seronegative Ocular Myasthenia

Treatment-refractory ophthalmoparesis/plegia among particularly the prepubertal group of juveniles with AChR-Ab negative MG or MuSK-Ab negative MG, may raise the possibility of a congenital myasthenic syndrome (CMS). CMS usually manifests with features of fatigable ocular or generalized muscle weakness at birth or within the first year of life, and often with a family history of a similar phenotype (47). However, pathogenic variations in several CMS genes may manifest in childhood (*CHNRE*; *COLQ*; *DOK7; GFPT1; RAPSN)*, adolescence (*DPAGT1*), or even in adulthood (*CHRNA1*; *CHRNE*; *DOK7; GFPT1; RAPSN*) (47). Most of these CMS are accompanied by additional features such as dysmorphism (*CHRNA1*), or limb-girdle pattern of weakness (*GFPT1*; *GMPPB; DGPAGT1*) without EOM weakness or ptosis. Pathogenic gene variants in a few CMS genes may rarely cause diagnostic confusion with “treatment resistant ocular ± generalized myasthenia”; pathogenic variants in *CHRNE1* have been reported to present after infancy with mild ptosis or ophthalmoplegia and respond to cholinesterase inhibitors; *DOK7* pathogenic variants may present with limb-girdle weakness and ptosis; occasional pathogenic variants in *RAPSN* may cause fluctuating ptosis with/without generalized fatigability (36, 47). Although pathogenic variants in *COLQ* usually cause severe early onset axial weakness with sparing of EOMs, some cases may have later onset, milder disease with variable occurrence of ophthalmoplegia and ptosis; these patients do not respond to cholinesterase inhibitors (36).

#### Treatment-Resistant Ophthalmoplegia and Definitions

Myasthenic involvement of the EOMs, similar to non-ocular muscles, is expected to respond to immunosuppressive therapies (38). However, in 2007, we first highlighted the occurrence of chronic treatment-resistant ophthalmoparesis (or ophthalmoplegia) in a subset of patients with MG from South Africa, whereas their non-ocular muscles responded to immune therapies. Treatment-resistant ophthalmoplegia occurred more frequently in those with younger onset (<20 years), AChR-Ab positive MG, and in individuals with African genetic ancestry (48). Subsequently, cross-sectional data from different pediatric centers across South Africa showed that up to 30% of the children attending hospital-based clinics remained with degrees of ophthalmoplegia after several years of immune therapies, irrespective of whether they had ocular-only or generalized MG (11).

Although complete ophthalmoplegia (also referred to as “eyeball fixation”) (6) is mentioned in juvenile cohorts from Asia, and elsewhere, it is frequently not quantified. Nevertheless, a Korean cohort of childhood-onset ocular MG (onset before 15 years and follow-up > 6 months) reported that only 29% (of 24 cases) improved in response to treatment with pyridostigmine and prednisone and 10% of patients remained with total ophthalmoplegia; only 50% were treated with prednisone and pyridostigmine (40) (Table 2). Treatment-resistant ophthalmoplegia was also reported in cohorts from Italy (3 of 19, 15%) and Canada (1 of 25, 4%) comprising either childhood-onset generalized or ocular MG and was frequently treated with immunosuppressive therapies and thymectomies (13, 49). Children with ocular MG from the USA (*n* = 21; followed for 2 years) showed “limitation of ductions” in 81% and complete resolution of myasthenic signs occurred in only 19%, although only a third had received steroids (42). Treatment resistance requiring oculoplastic surgery was reported in 6% of mainly Caucasian juveniles in another US cohort (19).

Taken together, younger African and Asian children with myasthenic involvement of EOMs appear to be at greater risk of developing treatment-resistant ophthalmoplegia (11, 29, 40). It is important to note that adult-onset MG cases, irrespective of ocular only MG or generalized MG, may develop treatment-resistant ophthalmoplegia including those with MuSK-Ab positive MG, triple seronegative MG, and older men with AChR-Ab positive MG (38, 50–52).

**Figure 1:**
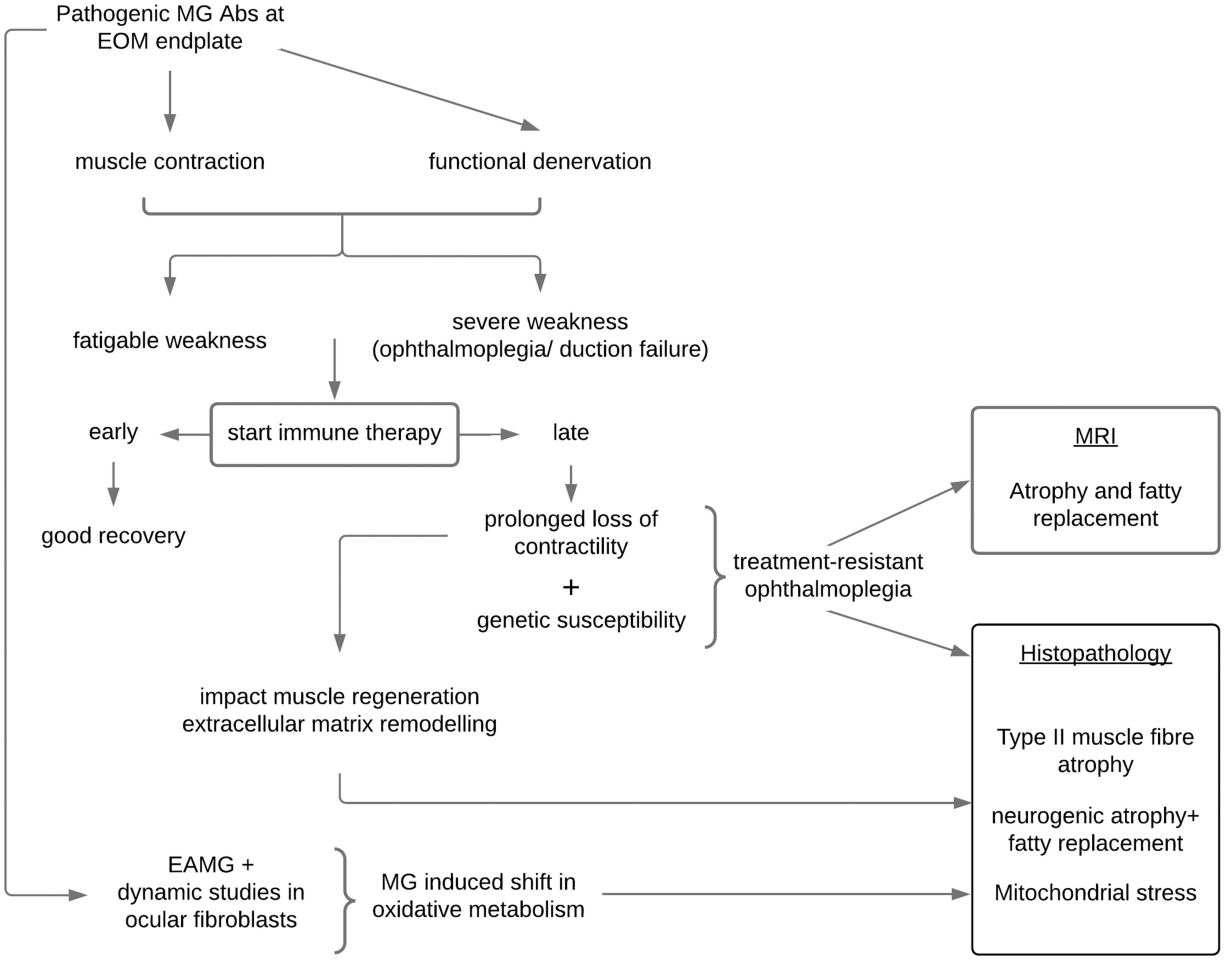
Proposed mechanisms in the development of treatment-resistant ophthalmoplegia in susceptible patients with myasthenia gravis. EOM, extraocular muscles; MRI, magnetic resonance imaging of the orbit; EAMG, experimental autoimmune myasthenia gravis; Abs, antibodies.

Presently, there is no definition for treatment-resistant or refractory ophthalmoplegia in MG. Definitions related to refractory generalized MG do not apply as patients with ophthalmoplegia (± ptosis) may experience substantial visual disability while the remaining non-ocular muscles may not be severely weak. In addition, refractoriness in generalized disease often requires documentation of treatment non-responsiveness and failure to prevent severe generalized MG weakness or crisis after trying several immune therapies for 12 to 24 months (53, 54), whereas observations suggest EOMs are vulnerable to shorter periods of inactivity due to functional denervation. Therefore, the definition of treatment-resistant ophthalmoplegia cannot be conservative as waiting for long periods in this setting may be counterproductive and contribute to muscle atrophy (Figure 1). Longitudinal observations of new patients with MG with persistent ophthalmoparesis/plegia and the timing of their resolution (or not) to immune therapy suggest that a signal for treatment non-responsiveness in most cases is evident around 6–7 months (38). However, another scenario occurs in which patients with MG may only manifest treatment-resistant ophthalmoplegia later, even after initially showing treatment responsiveness of their EOMs; in these cases, usually in the context of generalized disease, we noted that a critical event (infection; abrupt non-compliance) resulted in a relapse of MG and ophthalmoplegia with ongoing persistent non-responsiveness of the EOMs while the non-ocular muscles responded to the re-introduction/adjustments of MG therapies. We postulate that these events may have triggered critical biological pathways (see below) (39).

The clinical examination in patients with MG with chronic treatment-resistant ophthalmoplegia may also vary; some patients show an initial brief quiver movement as the saccadic movement is initiated before the eye stops short of its reduced trajectory, or brief lid twitches with attempted upgaze after a period of downgaze may be observed. However, after years of treatment-resistant ophthalmoplegia, the EOMs of some patients with MG show very limited and slow movements, and in some, there is no observable movement at all. When there is complete ophthalmoplegia, forced duction testing by an ophthalmologist may distinguish whether an apparently “fixed” eyeball can move through its trajectory; this can distinguish between severe eye muscle paralysis, where there is no mechanical restriction to forced EOM duction, and a restrictive force which prevents ocular movement (infiltration or fibrosis). In the setting of concomitant thyroid eye disease, the EOMs would show limited mechanical movement (39).

### Genetic Differences of Juvenile MG by Race/Population

#### Human Leukocyte Antigen Genes

The HLA region on chromosome 6 was the first genetic region, encompassing various class I (*HLA-A, HLA-B*) and class II genes (*HLA-DR, HLA-DQ*), shown to associate with MG (55). These HLA genes encode molecules that present antigens to CD4+ T helper cells which are necessary to mount an adaptive immune response specific to foreign pathogens [reviewed in Nel et al. (56)]. Although many HLA association studies have been performed in adults with MG, those in juveniles and children are sparse, but may suggest that juvenile and/or ocular MG may have a distinct immunological basis in certain populations (Table 3). For example, children from Norway showed an association with DRB1^*^04 (61) whereas those from Asia were associated with DRB1^*^09. Childhood-onset ocular MG in Japanese and Chinese children, who were predominantly AChR-Ab negative, have shown reasonably consistent HLA-B^*^4601; DRB1^*^0901 associations. Nel et al., found a higher frequency of functional variants in the *HLA-DRB1* region in a selected sample of African juveniles with treatment-resistant ophthalmoplegia (see below) compared to MG cases who responded to therapy (65), as well as the closely linked *HLA-DPB1* region (2). Preliminary results suggest that “low expression” HLA-DPB1^*^105:01 genotypes, which were also more common in African controls compared to European controls, associated with African juveniles with treatment-resistant ophthalmoplegia (2).

**Table 3:** Human leukocyte antigen (HLA) associations in juvenile myasthenia gravis by racial ancestry or geographical area.

**Type**	**Symptom onset, y**	**HLA gene associations**	**Geographical area**
Pre-pubertal MG	<10 <12	-DR9; Dw13 -DRB1^*^0404	Japan (57, 58) China (59, 60) Norway (61)
Post-pubertal MG	12-18	-B^*^08	Norway (61)
Juvenile MG	<15 <20	-DRB1^*^0901 -DRB4^*^0101	China (59) Japan (58, 62)
Ocular MG	<15 <18	-DQA1^*^0302;	China (63)
		DQB1^*^0303:02	Japan (62)
		-DRB1^*^1302;	
		DQA1^*^0102;	
		DQB1^*^0604	
		-DRB1^*^0901;	
		DQA1^*^0301;	
		DQB1^*^0303	
		-B^*^4601;	China (59, 64)
		DRB1^*^0901	
		-B^*^4601;	
		DRB1^*^0403	

MG to myasthenia gravis. Ocular MG when MG has been confined to ocular muscles for >2 years. Y, years. Both serological and molecular HLA typing methods were considered. HLA alleles derived from molecular typing are denoted with an asterix (*) e.g., DRB1

* *0901 is the gene for the serotype DR9. For more detail on the curation of HLA studies see Nel and Heckmann (56)*.

#### Pathogenic Mechanisms of Treatment-Resistant Ophthalmoplegia in MG

Our current hypothesis is that in a genetically susceptible individual, treatment-resistant ophthalmoplegia is likely the result of a complex network of dysregulated genes “activated” within the context of MG (39). Against this backdrop and together with a critical period of loss of contractility in the EOMs, muscle atrophy-pathways and mitochondrial metabolic pathways are not able to maintain normal homeostasis, and the paralysis of the EOMs may enter an irreversible phase of mitochondrial stress, EOM atrophy, and fat replacement (66, 67). Importantly, these histopathological changes may not be peculiar to MG, but rather to EOMs (more than other skeletal limb muscles) being particularly vulnerable to atrophy when contractility is compromised for a critical period irrespective of the cause (67). Similar to the EOM histopathological findings, imaging of the EOMs in patients with MG with chronic refractory ocular symptoms, found evidence of muscle atrophy and fatty replacement (52). Interestingly, fatty replacement with larger muscle volume was evident in the EOMs of a pilot case series (feasibility study) earlier in their disease course (68), whereas those with a longer disease duration showed muscle atrophy (69).

Gene expression studies in the EOMs of experimentally induced MG in rodents have also pointed to altered oxidative metabolism (70) which may in turn impact on EOMs maintaining high firing rates and generating contractile force (Figure 1). Poor muscle force generation affects mitochondrial biogenesis and triggers muscle atrophy signaling pathways (71, 72) all of which have been shown to be relevant in MG *in-vitro* modeling (73). The patient developing treatment-resistant ophthalmoplegia may be genetically susceptible to the induction of these “dysregulated” pathways only when they develop MG and possibly enter an irreversible stage when not treated early enough.

Although genetic studies have been limited due to the rarity of these patients, candidate gene approaches in juvenile AChR-Ab positive generalized patients with MG with the treatment-resistant ophthalmoplegic phenotype showed associations with regulatory variants in both the *DAF* (-*198 C*>*G*) and *TGFB1* (-*387 C*>*T*) genes (74, 75). However, these genetic associations did not account for many of the cases.

An unbiased genome-wide analysis in a highly selected enriched group of juveniles with treatment-resistant ophthalmoplegic MG compared to a matched control group of young myasthenic responders (extreme phenotype approach) identified several genes by their putative functional gene variant burden, which associated with ophthalmoplegic cases (2). Prioritizing these genes by their expression levels in muscle showed they converged on muscle atrophy signaling and myosin II function pathways (2). These predictions were validated in gene expression studies using orbital muscle biopsies of MG cases compared to an independent control group without MG, pointing to dysregulated muscle networks in the ophthalmoplegic MG cases involving muscle atrophy and/or contractility as well as oxidative metabolism gene pathways (76). These pathways identified by gene variant burden, showed significant dysregulated correlations (which differed from controls) with known MG genes/pathways (70, 73), highlighting the importance of the MG context. The unmet need is developing a prognostic biomarker for the early detection of these cases.

## Concluding Remarks

In juveniles with myasthenia, there are phenotypic differences amongst different populations in their ages at presentation, the proportions of ocular vs. generalized manifestation of MG, and in the treatment responsiveness of EOMs to immune therapies. Although ocular MG in younger children is often benign and self-limiting, indications are of genetically susceptible individuals who require a more aggressive approach with immune therapy to avoid chronic visual morbidity. There is a critical need for a prognostic biomarker to guide treatment approaches. In addition, clear knowledge gaps were identified; there is a lack of standardized use of descriptions of eye muscle involvement in juveniles with MG, and poor descriptions of their responsiveness (or lack thereof) to immune therapies. The field will benefit from a collaborative response to these research gaps.

## Author Contributions

JH conceived the idea and wrote the first draft and prepared Table 2. AS performed the literature review and editorial input and prepared Table 1. TE and MN provided editorial input and prepared the figure and Table 3. All authors contributed to the article and approved the submitted version.

## Funding

JH and TE received funding from the South African (SA) National Research Funding Agency and MN is the recipient of a CReATe scholars award and a Carnegie Developing Emerging Academic Leaders (DEAL) award. This publication was made possible (in part) by a grant from Carnegie Corporation of New York and a L'Oréal-UNESCO For Women in Science South African Young Talents Award. AS is supported by an educational grant from Life Healthcare.

## Conflict of Interest

The authors declare that the research was conducted in the absence of any commercial or financial relationships that could be construed as a potential conflict of interest.

## Publisher's Note

All claims expressed in this article are solely those of the authors and do not necessarily represent those of their affiliated organizations, or those of the publisher, the editors and the reviewers. Any product that may be evaluated in this article, or claim that may be made by its manufacturer, is not guaranteed or endorsed by the publisher.
